# A Robust Predictive Resource Planning under Demand Uncertainty to Improve Waiting Times in Outpatient Clinics

**DOI:** 10.1177/0972063417727627

**Published:** 2017-11-18

**Authors:** Jyoti R. Munavalli, Shyam Vasudeva Rao, Aravind Srinivasan, Usha Manjunath, G. G. van Merode

**Affiliations:** 1 Research Associate, Maastricht University Medical Centre, Maastricht, The Netherlands; 2 Cofounder, President and CTO, Forus Health, Bangalore, Technical Director, Maastricht University Medical Centre, Maastricht, The Netherlands; 3 Administrator, Aravind Eye Care, Madurai, Tamil Nadu, India; 4 Professor, Institute of Health Management Research, Bangalore, Karnataka, India; 5 Professor of Logistics and Operations Management in Health Care, Maastricht University Medical Centre, Maastricht, The Netherlands

**Keywords:** Predictive resource planning, waiting time, Takt time management, reaction time, outpatient clinic, demand-supply

## Background and context:

Resource planning is performed ahead of time within outpatient clinics (OPC). Due to local control of operations (department-centric decision-making) and limited resources, OPCs cannot handle high variability and uncertainty in demand. There is always a difference between planning and reality, and this leads to operational problems such as excessive waiting times. The OPCs often react to the situation when problems are encountered and reaction times play an important role in determining patient waiting times.

## Objectives:

To propose a predictive resource planning that incorporates variability in the short term with the OPC-wide perspective, not department-centric.

## Methodology:

The process and patient data were collected from the OPC under study by observation, interviews and from the records of the hospital management information system. A resource planning model (RPM) was developed that matched resources according to demand in short term. A mathematical model with outputs resource plan for a day was formulated utilizing Takt time (the average time a patient needs to move out of the OPC system) management that is used in Toyota Production System (TPS), to allocate resources to all the departments. Using a Discrete Event Simulation Model, the effects of predictive resource planning with different reaction times on waiting times and cycle times were analyzed. The resource plans were implemented in the OPC of Aravind Eye Hospital, Madurai, Tamil Nadu, India, that has high patient volumes and random patient arrivals.

## Results and discussion:

The simulation and implementation results indicate that predictive resource planning is robust and improves waiting times, and cycle times in OPCs. Study findings confirm that the predictive planning model reduces the average waiting time by 43.4 per cent during simulation and by 41.1 per cent during its implementation. The reduction in standard deviations in waiting times indicate reduction of unregulated waiting times. The OPC scheduled 28 resources throughout the day, whereas with predictive resource planning, the number of resources varied between a minimum of 12 to a maximum around 30–34 resources.

## Conclusions:

The OPCs currently match demands to their supply, while matching resources to varying demand in short term; throughout the OPC (all departments) improves patient flow, and minimizes waiting time and cycle time. Previously, Takt time management (TTM) has applied to systems with even and stable demand; in this study, it has been applied to stochastic demand.

## Implications:

This planning model helps the management to identify resource requirements: types of resources and number of resources, for the future demand growth and expansion. It can probably be extended to general hospitals by considering their demand forecast, precedence constraints and workflow complexities.

## References

[bibr1-0972063417727627] Activity-Report. (2014–2015). Aravind eye care system (p. 76). Madurai. Retrieved 8 4 2017, from http://www.aravind.org/default/researchnewcontent/annualreports

[bibr2-0972063417727627] Alvarez Roberto dos Reis Antunes José Antonio Valle Jr. (2001). Takt-time: Concepts and context in Toyota Production System. Gestão & Produção, 8(1), 1–18.

[bibr3-0972063417727627] Andersen Michael Moesgaard Poulfelt Flemming (2014). Beyond strategy: The impact of next generation companies. New York, NY: Routledge.

[bibr4-0972063417727627] Brilliant Larry Brilliant Girija (2007). Aravind: Partner and social science innovator (Innovations case discussion: Aravind eye care system). Innovations: Technology, Governance, Globalization, 2(4), 50–52.

[bibr5-0972063417727627] Cayirli T. Veral E. (2003). Outpatient scheduling in health care: A review of literature. Production and Operations Management, 12(4), 519–549.

[bibr6-0972063417727627] Chan H. Y. Lo S. M. Lee L. L. Y. Lo W. Y. L. Yu W. C. Wu Y. F. … Chan J. T. S. (2014). Lean techniques for the improvement of patients’ flow in emergency department. World Journal of Emergency Medicine, 5(1), 24–28. DOI: 10.5847/wjem.j.issn.1920-8642.2014.01.004 25215143 PMC4129868

[bibr7-0972063417727627] Chaudhary Bhupinder Modi G. Ashwin Reddy Kalyan (2012). Right to sight: A management case study on Aravind Eye Hospitals. ZENITH International Journal of Multidisciplinary Research, 2(1), 447–457.

[bibr8-0972063417727627] Curry Guy L. Feldman Richard M. (2010). Manufacturing systems modeling and analysis (2nd ed.). Berlin; Heidelberg: Springer-Verlag.

[bibr9-0972063417727627] Day Robert W. Dean Matthew D. Garfinkel Robert Thompson Steven (2010). Improving patient flow in a hospital through dynamic allocation of cardiac diagnostic testing time slots. Decision Support Systems, 49, 463–473. DOI: 10.1016/j.dss.2010.05.007

[bibr10-0972063417727627] Duggan Kevin J. (2002). Creating mixed model value steams—Practical lean techniques for building to demand. Boca Raton, FL: Productivity Press.

[bibr11-0972063417727627] Edward G. M. Das S. F. Elkhuizen S. G. Bakker P. J. M. Hontelez J. A. M. Hollmann M. W. … Lemaire L. C. (2008). Simulation to analyse planning difficulties at the preoperative assessment clinic. British Journal of Anaesthesia, 100(2), 195–202. 18211993 10.1093/bja/aem366

[bibr12-0972063417727627] Eswaramoorthi M. Kathiresan G. R. Jayasudhan T. J. Prasad P. S. S. Mohanram P. V. (2012). Flow index based line balancing: A tool to improve the leanness of assembly line design. International Journal of Production Research, 50(12), 3345–3358. DOI: 10.1080/00207543.2011.575895

[bibr13-0972063417727627] Fekete Milan Hulvej Jaroslav (2013). “Humanizing” Takt time and productivity in the labor-intensive manufacturing systems. Paper presented at the Knowledge Management and Innovation Management, Knowledge and Learning International Conference, Croatia.

[bibr14-0972063417727627] Gupta Diwakar Denton Brian (2008). Appointment scheduling in health care: Challenges and opportunities. IIE Transactions, 40, 800–819.

[bibr15-0972063417727627] Harper Paul R. (2002). A framework for operational modelling of hospital resources. Health Care Management Science, 5(3), 165–173. 12363044 10.1023/a:1019767900627

[bibr16-0972063417727627] Hassan M. H. Zaqhloul A. A. Mokhtar S. A. (2005). The probability distribution of attendance to hospital emergency units for school students in Alexandria. The Journal of the Egyptian Public Health Association, 80(1–2), 127–151. 16922150

[bibr17-0972063417727627] Hopp Wallace J. Lovejoy William S. (2012). Hospital operations: Principles of high efficiency health care (FT Press Operations Management). New Jersey: FT Press.

[bibr18-0972063417727627] Hopp Wallace J. Spearman Mark L. (1996). Factory physics: Foundations of manufacturing management. Irwin: The McGraw-Hill.

[bibr19-0972063417727627] Huang X. M. (1994). Patient attitude towards waiting in an outpatient clinic and its applications. Health Services Management Research: An official Journal of the Association of University Programs in Health Administration / HSMC, AUPHA, 7(1), 2–8. 10.1177/095148489400700101 10133292

[bibr20-0972063417727627] Jacobs J. H. Etman L. F. P. van Campen E. J. J. Rooda J. E. (2003). Characterization of operational time variability using effective process times. IEEE Transactions on Semiconductor Manufacturing, 16(3), 511–520.

[bibr21-0972063417727627] Karlis Dimitris Xekalaki Evdokia (2005). Mixed Poisson distributions. International Statistical Review, 73, 35–58. Retrieved from http://www.jstor.org/stable/25472639

[bibr22-0972063417727627] Li Jia Zha Hongyuan (2006). Two-way Poisson mixture models for simultaneous document classification and word clustering. Computational Statistics and Data Analysis, 50(1), 163–180. DOI: 10.1016/j.csda.2004.07.013

[bibr23-0972063417727627] Liker J. K. (2004). The Toyota way: 14 management principles from the world’s greatest manufacturer. New York, NY: McGraw-Hill.

[bibr24-0972063417727627] Ludwig Martijn Van Frits Merode Groot Wim (2010). Principal agent relationships and the efﬁciency of hospitals. European Journal of Health Economics, 11(3), 291–304. DOI: 10.1007/s10198-009-0176-z 19655184 PMC2860099

[bibr25-0972063417727627] Mansdorf Bruce D. (1975). Allocation of resources for ambulatory care—A staffing model for outpatient clinics. Public Health Reports, 90(5), 393–401. 809787 PMC1437857

[bibr26-0972063417727627] Mehta Pavithra K. Shenoy Suchitra (2011). Infinite vision—How Aravind became the greatest business case for compassion. San Franciso: Berrett-Koehler.

[bibr27-0972063417727627] Molema J. J. W. Groothuis S. Baars I. J. Kleinschiphorst M. Leers E. G. E. Hasman A. van Merode G. G. (2007). Healthcare system design and part time working doctors. Health Care Management Science, 10, 365–371. DOI: 10.1007/s10729-007-9032-9 18074969

[bibr28-0972063417727627] Natchiar G. Thulasiraj R. D. Sundaram R. Meenakshi (2008). Cataract surgery at Aravind Eye Hospitals: 1988–2008. Community Eye Health, 21(67), 40–42. 19030127 PMC2580063

[bibr29-0972063417727627] Nguyen T. B. T. Sivakumar A. I. Graves S. C. (2015). A network flow approach for tactical resource planning in outpatient clinics. Health Care Management Science, 18(2), 124–136. DOI: 10.1007/s10729-014-9284-0 24879403

[bibr30-0972063417727627] Ortiz Chris A. (2006). Kaizen assembly: Designing, constructing and managing a lean assembly line. Boca Raton, FL: CRC Press.

[bibr31-0972063417727627] Pillay D. I. Ghazali R. J. Manaf N. H. Abdullah A. H. Bakar A. A. Salikin F. … Ismail W. I. (2011). Hospital waiting time: The forgotten premise of healthcare service delivery? International Journal of Health Care Quality Assurance, 24(7), 506–522. DOI: 10.1108/09526861111160553 22204085

[bibr32-0972063417727627] Rangan V. K. Thulasiraj R. D. (2007). Making sight affordable (Innovations case narrative: Aravind eye care system). Innovations: Technology, Governance, Globalization Fall, 2(4), 35–49.

[bibr33-0972063417727627] Rother Mike (2009). Toyota Kata: Managing people for improvement, adaptiveness and superior results. New Delhi: McGraw-Hill Professional.

[bibr34-0972063417727627] Sandanayake Y. G. Oduoza C. F. (2009). Dynamic simulation for performance optimization in just-in-time-enabled manufacturing processes. International Journal of Advanced Manufacturing Technology, 42(3–4), 372–380. DOI: 10.1007/s00170-008-1604-4

[bibr35-0972063417727627] Smith James MacGregor Tan Baris (2013). Handbook of stochastic models and analysis of manufacturing system operations (Vol. 192). New York, NY: Springer-Verlag.

[bibr36-0972063417727627] van Merode Godefridus G. Groothuis Siebren Hasman Arie (2004). Enterprise resource planning for hospitals. International Journal of Medical Informatics, 73(6), 493–501. DOI: 10.1016/j.ijmedinf.2004.02.007 15171978

[bibr37-0972063417727627] Vermeulen Ivan B. Bohte Sander M. Elkhuizen Sylvia G. Lameris Han Bakker Piet J. M. Poutre Han La (2009). Adaptive resource allocation for efficient patient scheduling. Artificial Intelligence in Medicine, 46(1), 67–80. 18845423 10.1016/j.artmed.2008.07.019

[bibr38-0972063417727627] Vissers J. M. H. Bertrand J. W. M. de Vries G. (2001). A framework for production control in health care organizations. Production Planning & Control: The Management of Operations, 12(6), 591–604.

[bibr39-0972063417727627] Voort M. M. Rouppe van der Merode F. G. van Berden B. H. (2010). Making sense of delays in outpatient specialty care: A system perspective. Health Policy, 97(1), 44–52. DOI: 10.1016/j.healthpol.2010.02.013 20347179

[bibr40-0972063417727627] Yurko Lynne C. Coffee Tammy L. Fusilero Jane Yowler Charles J. Brandt Christopher P. Fratianne Richard B. (2001). Management of an inpatient-outpatient clinic: An eight-year review. Journal of Burn Care & Research, 22(3), 250–254. 10.1097/00004630-200105000-00014 11403250

[bibr41-0972063417727627] Zhu Zhecheng BeeHoon Heng Teow KiokLiang (2012). Analysis of factors causing long patient waiting time and clinic overtime in outpatient clinics. Journal of Medical Systems, 36(2), 707–713. DOI: 10.1007/s10916-010-9538-4 20703659

